# Conserved Influenza Hemagglutinin, Neuraminidase and Matrix Peptides Adjuvanted with ALFQ Induce Broadly Neutralizing Antibodies

**DOI:** 10.3390/vaccines9070698

**Published:** 2021-06-25

**Authors:** Clara J. Sei, Mangala Rao, Richard F. Schuman, Luke T. Daum, Gary R. Matyas, Nimisha Rikhi, Kevin Muema, Alexander Anderson, Ousman Jobe, Kellie A. Kroscher, Carl R. Alving, Gerald W. Fischer

**Affiliations:** 1 Longhorn Vaccines and Diagnostics, Gaithersburg, MD 20878, USA; nr@lhnvd.com (N.R.); kevin@lhnvd.com (K.M.); kellie@lhnvd.com (K.A.K.); gwf@lhnvd.com (G.W.F.); 2 U.S. Military HIV Research Program, Walter Reed Army Institute of Research, Silver Spring, MD 20910, USA; mrao@hivresearch.org (M.R.); gmatyas@hivresearch.org (G.R.M.); aanderson@hivresearch.org (A.A.); ojobe@hivresearch.org (O.J.); calving@hivresearch.org (C.R.A.); 3 Antibody and Immunoassay Consultants, Rockville, MD 20850, USA; rfs@aicbiotech.com; 4 Longhorn Vaccines and Diagnostics, San Antonio, TX 78209, USA; longhorn-vandd@sbcglobal.net; 5 Oak Ridge Institute of Science and Education, Oak Ridge, TN 37831, USA; 6 Henry M Jackson Foundation for the Advancement of Military Medicine, Bethesda, MD 20817, USA

**Keywords:** peptides, influenza, ALFQ, liposomes, QS21 (also known as QS-21), universal vaccine, immune responses, broadly reactive antibodies, neutralizing antibodies, Th1/Th2 responses

## Abstract

A universal influenza candidate vaccine that targets multiple conserved influenza virus epitopes from hemagglutinin (HA), neuraminidase (NA) and matrix (M2e) proteins was combined with the potent Army liposomal adjuvant (ALFQ) to promote induction of broad immunity to seasonal and pandemic influenza strains. The unconjugated and CRM-conjugated composite peptides formulated with ALFQ were highly immunogenic and induced both humoral and cellular immune responses in mice. Broadly reactive serum antibodies were induced across various IgG isotypes. Mice immunized with the unconjugated composite peptide developed antibody responses earlier than mice immunized with conjugated peptides, and the IgG antibodies were broadly reactive and neutralizing across Groups 1 and 2 influenza viruses. Multi-epitope unconjugated influenza composite peptides formulated with ALFQ provide a novel strategy for the development of a universal influenza vaccine. These synthetic peptide vaccines avoid the pitfalls of egg-produced influenza vaccines and production can be scaled up rapidly and economically.

## 1. Introduction

Influenza is a major cause of respiratory infections around the world. The global influenza disease burden has been estimated to approach up to 1 billion infections, 3–5 million cases of severe disease and 300,000–500,000 deaths annually [1]. In addition, influenza pandemics occur at various times and cause varying levels of severity and death [2]. Seasonal influenza virus vaccine is the most utilized method to reduce the impact of influenza infection. The two major glycoproteins on the surface of influenza are hemagglutinin (HA) and neuraminidase (NA). Antibodies to HA are generally considered the key mediators of influenza immunity, and inhibition of HA activity has been the primary measure of vaccine effectiveness. Point mutations causing HA antigenic drift is the primary reason for variable and limited effectiveness of the seasonal influenza vaccine. While point mutations in the NA can occur, antigenic drift is lower than for HA among influenza A viruses [3,4]. In fact, a recent study showed that humans infected with influenza develop NA-reactive antibodies that are broadly reactive and protective [5].

New influenza vaccine strategies that focus on inducing antibodies to multiple key proteins could promote broad influenza immunity across seasonal and pandemic strains. In humans, HA-reactive antibodies constitute a subset of neutralizing antibodies that may be important in protection [6]. Neuraminidase drifts independently of HA, and immunity to NA has been correlated with protection [7]. The extracellular domain of Matrix 2 (M2e) protein is a highly conserved region in influenza A viruses [8]. Immunity to influenza induced by M2e may include both innate and adaptive immune responses and therefore has been considered an important target for developing a universal influenza vaccine [8,9]. The HA and NA peptides are highly conserved and located near or at the protein area where the receptor binding sites reside [10,11,12]. These epitopes classically are impacted by induction of antibodies through the adaptive immune system and a major target for influenza vaccines. To promote broad immunity across Groups 1 and 2 influenza viruses, structural mapping of the HA and NA proteins was performed to identify highly conserved epitope sequences. These peptide sequences were then combined and arranged as continuous sequences to form an HA and NA composite peptide antigen. The extracellular domain of the M2 matrix protein (M2e) is highly conserved and constitutes a set of epitopes that may function through different arms of the innate immune system [8,13]. Antibodies to M2e may not directly neutralize influenza viruses but utilize other immunologic mechanisms such as NK-mediated ADCC (antibody-dependent cellular cytotoxicity). The M2e epitope has also been considered an important target for developing a universal influenza A vaccine [8]. A composite M2e peptide was constructed by utilizing a highly conserved region of the M2e and then repeating a portion of the sequence [8,13]. A tetanus toxoid T-cell epitope was also added to the C-terminal end. These composite peptide vaccines containing highly conserved HA, NA and M2e epitopes were designed to induce broad immunity across seasonal and pandemic influenza strains. Since these peptide vaccines are also synthetically produced, they avoid propagation of the virus in eggs and therefore can be rapidly and efficiently manufactured.

It has been proposed that carrier proteins and adjuvants are necessary to elicit a strong immune response to peptide-based influenza vaccines [8,14]. Initial preliminary studies demonstrated that immunizing mice with these influenza composite peptides conjugated to a carrier protein, and with an adjuvant, induced broadly reactive, neutralizing antibodies across both Groups 1 and 2 influenza A virus strains (unpublished data). In addition, these composite influenza peptide/conjugate vaccines, administered with the saponin adjuvant Quil-A^®^, produced higher influenza IgG titers in mice against HA, NA and M2e, to include IgG1, IgG2a and IgG2b (unpublished data).

Previous studies demonstrated that liposomes could serve as vehicles both for adjuvants and for inducing antibodies and cellular immunity to immunogenic peptides [15]. Liposomes containing monophosphoryl lipid A (now referred to as Army Liposome Formulation, or ALF) were potent and effective for inducing antibodies and cell-mediated immunity both to conjugated and unconjugated HIV-1 peptides [15,16], and even for inducing antibodies to haptens for a candidate heroin vaccine [17,18] or for a combination heroin-HIV vaccine [19]. ALF-type adjuvants were also combined with different types of antigens in numerous phase 1 and phase 2 human clinical trials [20].

In 2015, liposomes with 55 mol % cholesterol and containing both monophosphoryl lipid A and QS21 saponin (ALFQ) were introduced as a novel and highly potent adjuvant formulation [21]. ALFQ is being used in three ongoing human phase 1 clinical trials for two different candidate malaria vaccines and a COVID vaccine and is projected for use in other types of candidate vaccines [22]. Because of the effectiveness of ALF-type adjuvants for induction of antibodies both to conjugated and unconjugated peptides, and because ALFQ has now been introduced as an adjuvant for candidate human vaccines, ALFQ was employed in the present study to explore its effectiveness as an adjuvant for inducing broad binding and neutralizing antibodies to composite influenza peptide antigens. In this report, we demonstrate that ALFQ adjuvant improved the immunogenicity of both conjugated and unconjugated composite influenza peptides and promoted the induction of a balanced Th1/Th2 response while increasing its neutralization breadth against both Group 1 and Group 2 influenza viruses.

## 2. Materials and Methods

### 2.1. Cell Culture

Madin-Darby canine kidney (MDCK) cells (ATCC, Manassas, VA, USA) were maintained in Eagle’s Minimal Essential Medium (EMEM) (Lonza, Basel, Switzerland) containing 5% fetal bovine serum (FBS) (Hyclone, Logan, UT, USA), 2 mM L-glutamine (Sigma-Aldrich, St. Louis, MO, USA), 1% sodium pyruvate and 1% non-essential amino acids (NEAA) (Thermo Fisher Scientific, Waltham, MA, USA), 1% amphotericin B (Sigma-Aldrich, St. Louis, MO, USA) and 0.1% gentamycin (Thomas Scientific, Swedesboro, NJ, USA).

### 2.2. Influenza Viruses

Several influenza viruses were obtained from the Influenza Reagents Resource (IRR) (Manassas, VA, USA), established by the Centers for Disease Control and Prevention (CDC), USA. Group 1 virus strains used include A/Michigan/45/2015 (H1N1) pdm09, A/California/07/2009 (H1N1) pdm09, and A/Wisconsin/505/2018 (H1N1) pdm09. Group 2 influenza virus strains used include A/Hong Kong/4801/2014 (H3N2), A/Victoria/361/2011 (H3N2) and A/Texas/50/2012 (H3N2). All viruses were propagated by infection of MDCK monolayers for 3 days at 37 °C and 5% CO_2_. Virus concentrations were determined by the standard tissue culture infectious dose (TCID_50_) assay on MDCK cells and aliquots were stored at −80 °C.

### 2.3. Influenza Peptides

Mice immunization studies were performed using a vaccine formulation of two composite influenza peptides, Flu Pep11 and Flu Pep5906, adjuvanted with ALFQ to evaluate humoral and cytokine immune responses. The individual and composite flu peptides were synthesized under a contract with Atlantic Peptides, Lewisburg, PA, USA. Flu Pep11, comprising HA epitopes (Flu Pep3 and Flu Pep6) and an NA epitope (Flu Pep10), was used in conjunction with Flu Pep5906, comprising M2e epitope and a tetanus T-cell epitope. The individual and composite peptides were used in immunoassays to evaluate binding and functional activities of anti-influenza antibodies.

### 2.4. Selection of Conserved HA and NA Epitopes

A primary amino acid multiple sequence alignment was performed using sequences derived from more than 2000 H1 and H3 influenza A field strains and representative H5 strains available from the literature and via public domain sequences deposited in NCBI at: www.ncbi.nlm.nih.gov/genomes/FLU/Database/nph-select.cgi?go=database (accessed on 13 March 2010) and through a personal database (L.T. Daum, unpublished). H3 and H1 vaccine and reference strains and H5 peptides representing clades 1, 1′, 2, and 3 were also included. Highly conserved peptide regions of influenza A hemagglutinin and neuraminidase were identified through multiple sequence alignments of several thousand strains. Sequences were obtained using the Influenza Virus Resource website (available at: http://www.ncbi.nlm.nih.gov/genomes/FLU/FLU.html (accessed on 13 March 2010)), and represent strains obtained from human, avian, and/or mammalian sources. Peptides were selected based on conserved HA and NA regions deduced from multiple sequence alignments (LaserGene, DNAStar Inc., Madison, WI, USA). The protein epitope 5′-GNLIAP-3′, corresponding to H3 amino acid positions 249–254, is a highly conserved region in H3 and H1 subtypes, while GNFIAP is highly conserved in H5. The GNLIAP region is partially exposed on the distal HA1 surface and is not located within previously proposed antigenic/immunodominant sites (A–E). Furthermore, WGVHHP, corresponding to H3 amino acid positions 180–185, is highly conserved in H3 and H1 subtypes. The corresponding region on H5, WGIHHP, differs by one residue-isoleucine. To demonstrate the utility of small, conserved influenza epitopes to induce immunity, several HA and NA conserved regions were conjugated to a protein carrier or unconjugated. These small, conserved influenza epitopes included sequence GNLFIAP, which is a novel combination of GN with both the L and F and then IAP. This contains all residues for H1, H3 and H5 subtypes.

**Figure 1:**
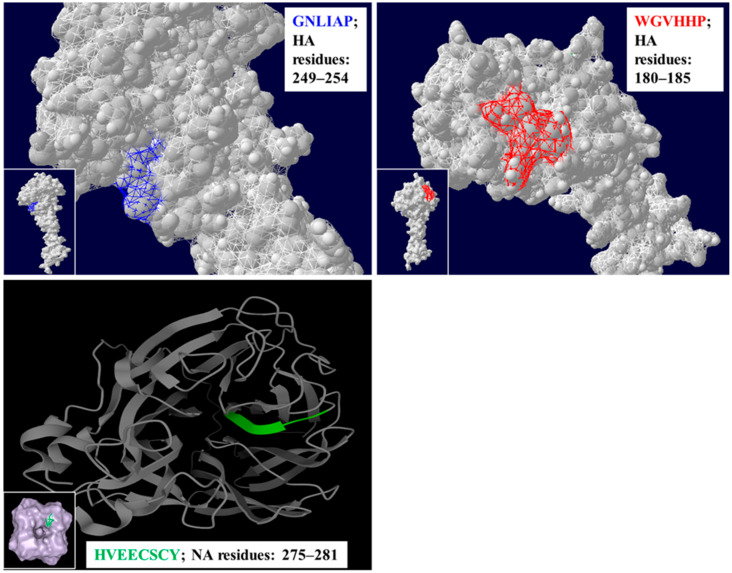
The panels on the top left and right show the structural positioning of the HA residues GNLIAP (in blue) and WGVHHP (in red), while the panel on the bottom shows the position of the NA residues HVEECSCY (in green) on a three-dimensional structure of H3N2 influenza virus.

Three-dimensional (3-D) folded HA and NA proteins were constructed based on the nearest homolog available in the Protein Sequencing Database with either Swiss PDF Viewer (Version 3.7) or Mol* viewer (PDB Protein Data Bank; www.rcsb.org (accessed on 13, March, 2010)) (Figure 1). Conserved residues observed in sequence alignments are depicted in the mature HA or NA proteins (Figure 1). For HA monomer, 3-D HA structure was derived from A/Wisconsin/67/2005 (H3N2) vaccine strain and constructed using Swiss PDF Viewer, while for the NA monomer, the 3-D NA structure was derived from A/Perth/16/2009 (H3N2) (GenBank 6BR6_A) and constructed using Mol* viewer (PDB Protein Data Bank; www.rcsb.org (accessed on 13, March, 2010)) (Figure 1). GNLFIAP, referred to as Flu Pep3, is a composite of GNLIAP (H3/H1 subtypes) and GNFIAP (H5 subtypes). WGVIHHP, referred to as Flu Pep6, is a composite of WGVHHP (H3/H1 subtypes) and WFIHHP (H5 subtype). GNLFIAPWGVIHHPHYEECSCY, referred to as Flu Pep11, is a composite that contains these three epitopes: GNLFIAP, WGVIHHP and HYEECSCY (Table 1). The NA monomer consists of a conserved epitope sequence with residues HYEECSCY (N1 subtype), referred to as Flu Pep10. This conserved epitope varies by a single amino acid between N1 and N2 neuraminidase with a Y (N1) and V (N2) at residue 276. Monoclonal antibodies to the HYEECSCY epitope were shown to neutralize both H1N1 and H3N2 influenza viruses (unpublished data).

### 2.5. Selection of Conserved M2e Epitopes

Because there is high M2e sequence conservation among human influenza viruses, M2e has been long proposed as an ideal universal influenza A vaccine candidate [9]. The highly conserved region of M1 and M2 (M2e sequence: MSLLTEVETPIRNEWGCRCNDSSD) was utilized to construct an M2e composite peptide that provides epitope repetition and a C-terminal T-cell epitope [8,13]. Selected epitopes were synthesized as a composite M2e vaccine with the C-terminal T-cell epitope and were utilized as a peptide vaccine with and without CRM conjugation. M2e Composite Peptide Vaccine—SLLTEVETPIRNEWGLLTEVETPIRQYIKANSKFIGITE, is referred to as Flu Pep5906 (Table 1).

### 2.6. Peptide Conjugation to CRM197

For Flu Pep11, thiol-ether chemistry was used to link cysteine residue to CRM197 (hereafter referred to as CRM) pre-labeled with 15 maleimides: 4 peptides (2.5 K) per CRM (Fina Biosolutions, Rockville, MD, USA). The conjugate was purified by dialysis (Hermanson G.T, Bioconjugate Techniques 3rd Edition) [23].

For Flu Pep5906, the peptide was labeled with a thiol linker N-Succinimidyl 3- (2-pyridildithio) propionate (SPDP) (CovaChem, Illinois, IL, USA) and then conjugated to CRM pre-labeled with 15 maleimides: 8 peptides (4.5 K) per CRM. The conjugate was purified by dialysis (Hermanson G.T., Bioconjugate Techniques, 3rd Edition) [23].

### 2.7. ALFQ Formulation with Composite Influenza Peptides

Dimyristoyl phosphatidylcholine (DMPC), dimyristoyl phosphatidylglycerol (DMPG) cholesterol (Chol), and synthetic monophosphoryl lipid A (MPLA, 3D-PHAD^®^) were purchased from Avanti Polar Lipids. DMPC and Chol were dissolved in chloroform, and DMPG and 3D-PHAD^®^ were dissolved in chloroform:methanol (9:1). QS21 was purchased from Desert King International and was dissolved in Sorensen PBS, pH 6.2 and filtered. For vaccine preparations adjuvanted with ALF containing QS21 (ALFQ), lipids were mixed in a molar ratio of 9:1:12.2:0.114 (DMPC:DMPG:Chol: 3D-PHAD^®^), dried by rotary evaporation followed by overnight desiccation, rehydrated by adding Sorensen PBS, pH 6.2, processed by microfluidization to form small unilamellar vesicles (SUV), and filtered [21]. QS21 was added to the SUVs to form ALFQ (DMPC:DMPG:Chol:MPLA:QS21; 9:1:12.2:0.114:0.044) [22].

Vaccines were prepared to contain either unconjugated Flu Pep11 and Flu Pep5906 or CRM-conjugated Flu Pep11 and Flu Pep5906. Vaccines containing CRM-conjugated peptides were prepared by adding 35, 17.5, 7, or 3.5 µg of each peptide to 186.4 µL of ALFQ (containing 751 µg/mL of 3D-PHAD) and then diluting to a 350 µL final volume with DPBS. Unconjugated flu peptide 11 and peptide 5906 were dissolved in 50% DPBS and then combined. Vaccines containing unconjugated peptides were prepared by adding 17.5, 7, or 3.5 µg of each peptide to 186.4 µL of ALFQ (containing 751 µg/mL of 3D-PHAD) and then diluting to a 350 µL final volume with DPBS. The vials were vortexed at slow speed for 1 min and then put on a roller for 15 min. The vials were stored at 4 °C prior to immunization. Each vaccine dose contained 20 µg 3D-PHAD, 10 µg QS21 (ALFQ), and 2.5, 1, or 0.5 µg of each peptide in 50 µL volume.

### 2.8. Mice Immunizations Using ALFQ Formulated Composite Peptides

All animal procedures were approved by the Institutional Animal Care and Use Committee (Sigmovir Biosystems Inc., Rockville, MD, USA, IACUC Protocol #71). Female Institute of Cancer Research (ICR-CD-1^®^) mice, 3–4 weeks of age, were obtained from Envigo (Indianapolis, IN, USA) for use in this study. Mice were housed in groups of 5 and maintained in a 12 h light–dark cycle with ad libitum access to feed and water. All mice were given a 2-week acclimatization period. Mice were randomly assigned to one of the 6 treatments (*n* = 5). The composite unconjugated or CRM-conjugated influenza peptides (Flu Pep11 + Flu Pep5906, in combination), formulated with ALFQ, were injected intramuscularly at doses of 0.5, 1, and 2.5 µg/mouse (*n* = 5 mice/dose/group). Mice were immunized on day 0 and booster immunizations were given on days 21 and 35. Submandibular bleeds were obtained on day 7 (pre-immunization), and days 7, 21, 28, 35, 42 and 56. Approximately 150–200 µL of blood were collected at each bleed and processed for serological testing.

### 2.9. Antisera ELISA: Detection of Antibodies That Bind to Influenza Peptides/Viruses

Serum anti-influenza levels were evaluated using peptides/antigens and live influenza viruses (H3N2 and H1N1). Next, 96-well NUNC MaxiSorp ELISA plates (Fisher Scientific, Pittsburg, PA, USA) were coated with peptides (1 µg/mL) and live influenza viruses (10^5^ TCID_50_ per well) in PBS at 100 µL per well for 18–24 h at room temperature (RT) and 2–8 °C, respectively. Antigen-coated plates were blocked with 3% normal goat serum (NGS, Southern Biotech, Birmingham, AL, USA) in PBS and subsequently washed with PBS-0.05% Tween 20 (PBS-T, Fisher Scientific, Pittsburg, PA, USA) using an ELx405 Automated Plate Washer (BioTek, Winooski, VT, USA). Serial dilutions of individual serum samples were added in duplicate. Anti-influenza antibodies were detected with Horse Radish Peroxidase (HRP)-conjugated, gamma-specific, goat anti-mouse IgG (Southern Biotech, Birmingham, AL, USA) or HRP-conjugated, IgG isotype-specific goat anti-mouse IgG1, IgG2a, IgG2b, IgG3 (Southern Biotech, Birmingham, AL, USA). TMB Substrate Solution (Fisher Scientific, Pittsburg, PA, USA) was added prior to being quenched with TMB STOP solution (Fisher Scientific, Pittsburg, PA, USA). The absorbance values (450 nM) of each well were obtained using a SpectraMax Plus Microplate Reader (Molecular Devices, Sunnyvale, CA, USA). Pre-immunization sera were used as negative controls. Antisera titers were calculated using the formula: EXP (((LN (b) − LN (a))/(d − c)) * (E − c) + LN (a)), where A = dilution giving absorbance above the desired titer point, B = dilution giving absorbance below the desired titer point, C = absorbance value at dilution A, D = absorbance value at dilution B, E = Titer Point selected (absorbance value of 0.5). Absorbance values consisted of OD readings at 450 nM.

### 2.10. Microneutralization Assay for Determination of Serum Neutralizing Antibodies

MDCK cells were seeded for 3–4 days in 96-well plates (Corning, Tewksbury, MA, USA). Pooled anti-influenza sera samples in quadruplets were heat-inactivated at 56 °C for 30 min and serially diluted twofold from a starting dilution of 1:40 in TPCK-trypsin medium in a 96-well plate. Then, 120 µL per well of influenza A viruses (H3N2 or H1N1) were added to each well containing 120 µL of diluted antisera and incubated for 2 h at 37 °C/5% CO_2_. This addition of an equal volume of virus into the anti-influenza sera samples further increased the antisera dilution twofold to a starting dilution of 1:80 and a final dilution of 1:5120. Confluent MDCK cell monolayers in 96-well plates were washed with PBS (Fisher Scientific, Pittsburg, PA, USA) using an ELx405 Automated Plate Washer (BioTek, Winooski, VT, USA) 10 min before the next step. After the 2 h incubation, 100 µL/well of each dilution of antisera-virus mixture were transferred to quadruplicate wells of pre-washed MDCK cell monolayers. Wells containing virus dilutions only and MDCK cells only were included in each plate as positive and negative controls for infectivity, respectively. The assay plates were incubated at 37 °C/5% CO_2_ for 4 days. After incubation, the plates were stained with Crystal Violet and scored by recording cytopathic effect (CPE) of virus on MDCK cell monolayer. Clear wells representing absence of MDCK cells were scored as positive CPE and indicated an absence of neutralization of viral replication by the antisera tested. Wells with a blue coloration representing presence of MDCK cells were scored as negative CPE and indicated neutralization of viral replication by the antisera tested. Neutralizing titers were reported as reciprocal of the highest dilution that showed complete protection of the MDCK cell monolayer, e.g., 1:2560 antisera dilution = 2560 neutralizing titer.

### 2.11. Preparation of Supernatants for Cytokine Determination

Spleens were collected from immunized mice on day 100 ± 7. Each spleen was removed and placed into a sterile 50 mL conical tube containing 25 mL of serum-free, high-glucose DMEM (Quality Biological Inc, Gaithersburg, MD, USA) supplemented with 2 mM L-glutamine (Corning Inc., Corning, NY, USA) and 50 µg/mL of gentamicin sulfate (Corning Inc., Corning, NY, USA) (SF-DMEM). Spleens were perfused with 10 mL of SF-DMEM. A single-cell suspension of splenocytes was prepared by gently pressing the spleen through a 60-mesh cell sieve and passed through a 70 µm Falcon cell strainer (Fisher Scientific, Hampton, NH, USA) into a 50 mL conical tube. The resulting cell suspension was pelleted by centrifugation and the supernatant discarded. Red blood cell lysis buffer (Sigma-Aldrich, St. Louis, MO, USA) was added to the pellet for 1 min at room temperature with gentle shaking and the cells were resuspended into 25 mL of SF-DMEM. The cells were again pelleted by centrifugation and, after removal of the supernatant, the cells were resuspended at a concentration of 4 × 10^6^ cells/mL in cDMEM (DMEM supplemented with 10% heat-inactivated FBS) (Cytiva, Marlborough, MA, USA), 2 mM L-glutamine, and 50 µg/mL of gentamicin sulfate. One mL of cell suspension was seeded into each well of a 24-well culture dish and 1 mL of Flu Pep11 or Flu Pep5906 in cDMEM were added to individual wells at final concentration of 30 µg/mL. Control wells received cDMEM alone. The cells were incubated in a humidified atmosphere at 37 °C/5% CO_2_ for 68–72 h. Cultures were harvested and centrifuged. Cell-free supernatants were aliquoted and stored at −20 °C until evaluated for cytokine concentrations. Cytokines and chemokines were assayed using a Quansys Biosystems multiplex system Q-PlexTM Array (Logan, UT, USA), according to the instructions of the manufacturer.

### 2.12. Statistical Analysis

Data was analyzed using GraphPad Prism 9 (San Diego, CA, USA). Antisera titer results were expressed as mean (±standard deviation and/or standard error of mean) from *n* = 5 mice per dose/group with significance threshold set at *p* < 0.05 using the two-way ANOVA with a Šidák correction for multiple comparisons. Statistical significance in neutralization titer results was evaluated using a Student *t*-test. Cytokine results were evaluated using the two-way ANOVA with a Šidák correction for multiple comparisons and significance threshold also set at *p* < 0.05.

## 3. Results

### 3.1. Antisera Titers on Individual and Composite Influenza Peptides

**Figure 2:**
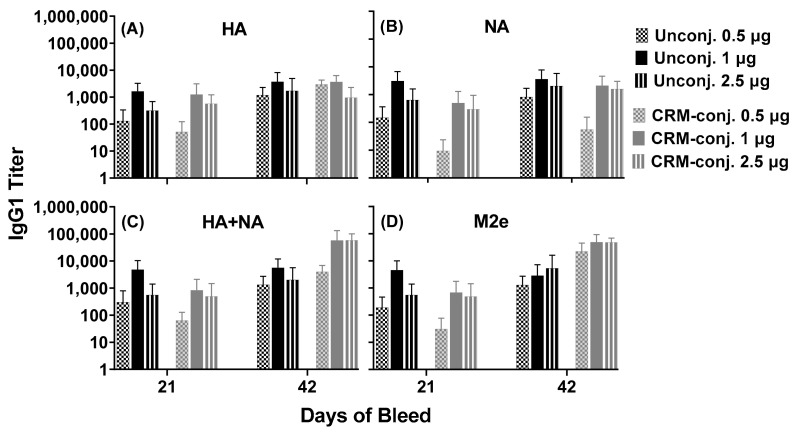
Serum antibody responses across conserved influenza epitopes in mice immunized with unconjugated or CRM-conjugated composite peptide (HA, NA and M2e) vaccine at 0.5, 1 and 2.5 µg/mouse, and formulated with ALFQ. Day 0, 21 and 42 sera samples were analyzed by ELISA. Panels (**A**,**B**), IgG1 titers (1:100 dilution) on individual peptides HA (Flu Pep3) and NA (Flu Pep10), and Panels (**C**,**D**), on composite peptides HA+NA (Flu Pep11) and M2e (Flu Pep5906), respectively. No significant differences were observed between the two groups as determined by ANOVA. Data are expressed as mean ± standard deviation (SD).

Unconjugated and CRM-conjugated universal composite influenza peptides (Flu Pep11 + 5906) (HA, NA and M2e) formulated with ALFQ were immunogenic in mice at all 3 doses tested (Figure 2). Isotype-specific antibody responses to HA, NA and M2e peptides for pre-immunization sera from all mice groups were undetectable. Serum IgG1 antibodies targeting highly conserved influenza HA, NA and M2e epitopes were induced for both unconjugated and CRM-conjugated groups 21 days post primary immunization. By Day 42, the serum IgG1 antibodies had increased for both unconjugated and CRM-conjugated peptides following the second boost on day 35.

Overall, the serum IgG1 antibodies for the unconjugated and CRM-conjugated peptides in the two dose groups (1 and 2.5 µg/mouse), demonstrated robust responses to HA, NA and M2e (titers ranging from 1000–100,000) on day 42. No significant differences were observed between the unconjugated and CRM-conjugated groups. Serum IgG2a and IgG3 antibody responses in both groups at all doses tested were negligible (data not shown).

**Figure 3:**
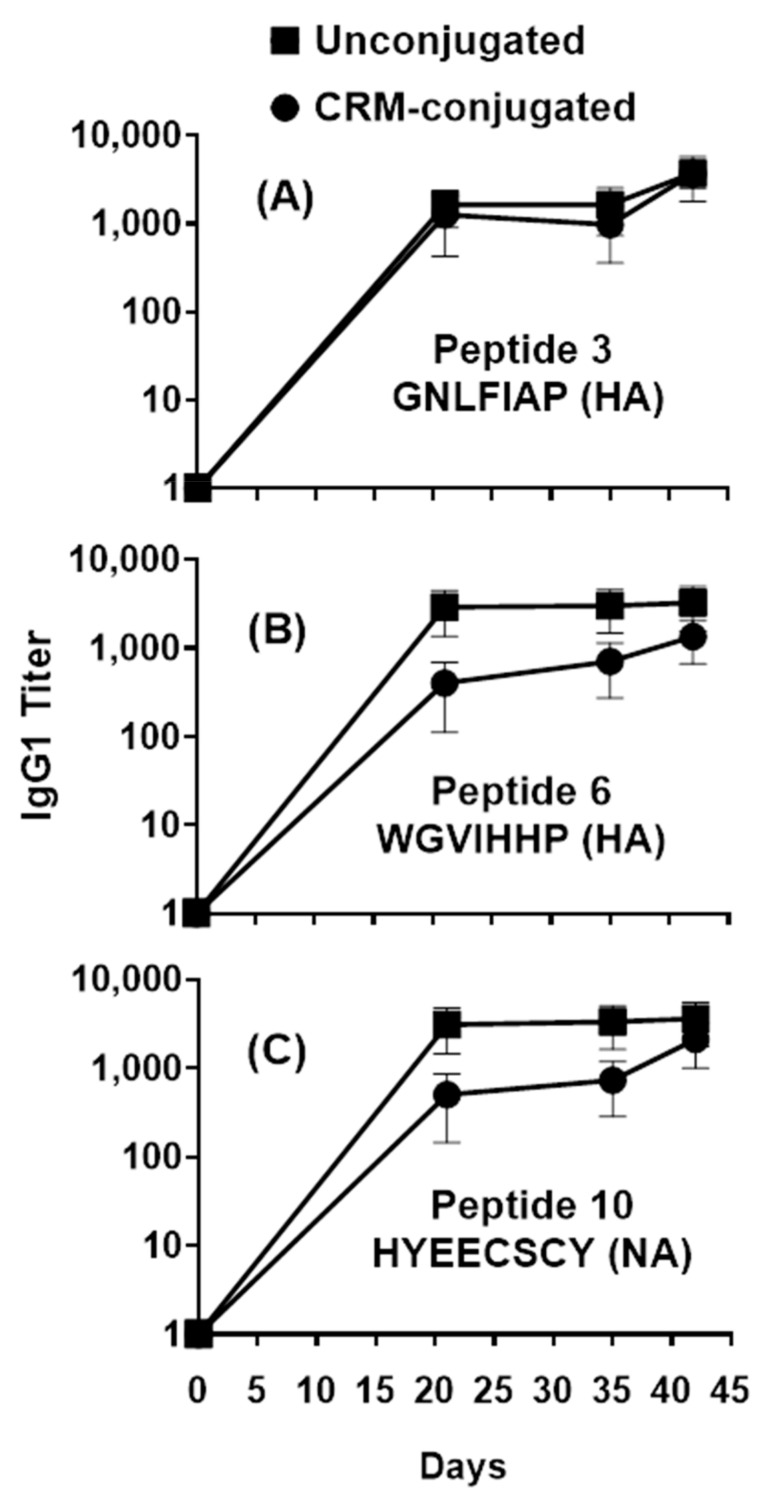
Profile of serum IgG1 responses across individual HA and NA peptides in mice immunized with 1µg of composite influenza peptide (HA, NA and M2e) vaccine, unconjugated or CRM-conjugated, and formulated with ALFQ. Day 0, 21, 35 and 42 sera samples were analyzed by ELISA. Panels (**A**) and (**B**), IgG1 titers (1:100 dilution) on Flu Pep3 and Flu Pep6 (both HA), respectively, and Panel (**C**), on Flu Pep10 (NA). Data are expressed as mean ± standard errors (SEM). Overall, the antibody responses on day 21 were significantly higher (ANOVA, *p* = 0.0435) for unconjugated peptides compared to CRM-conjugated peptides, while their differences on day 42 were not statistically significant.

Notably, a closer focus on the 1 µg/mouse dose groups revealed that compared to the CRM-conjugated peptides, IgG1 antibody responses for unconjugated peptides were higher across the individual HA (Flu Pep3 and Pep6) and NA (Flu Pep10) conserved epitopes on both days 21 and 42 (Figure 3). IgG1 antibody responses for unconjugated mice were higher early (on day 21) across the composite HA+NA (Flu Pep11) and M2e (Flu Pep5906) conserved epitopes.

**Figure 4:**
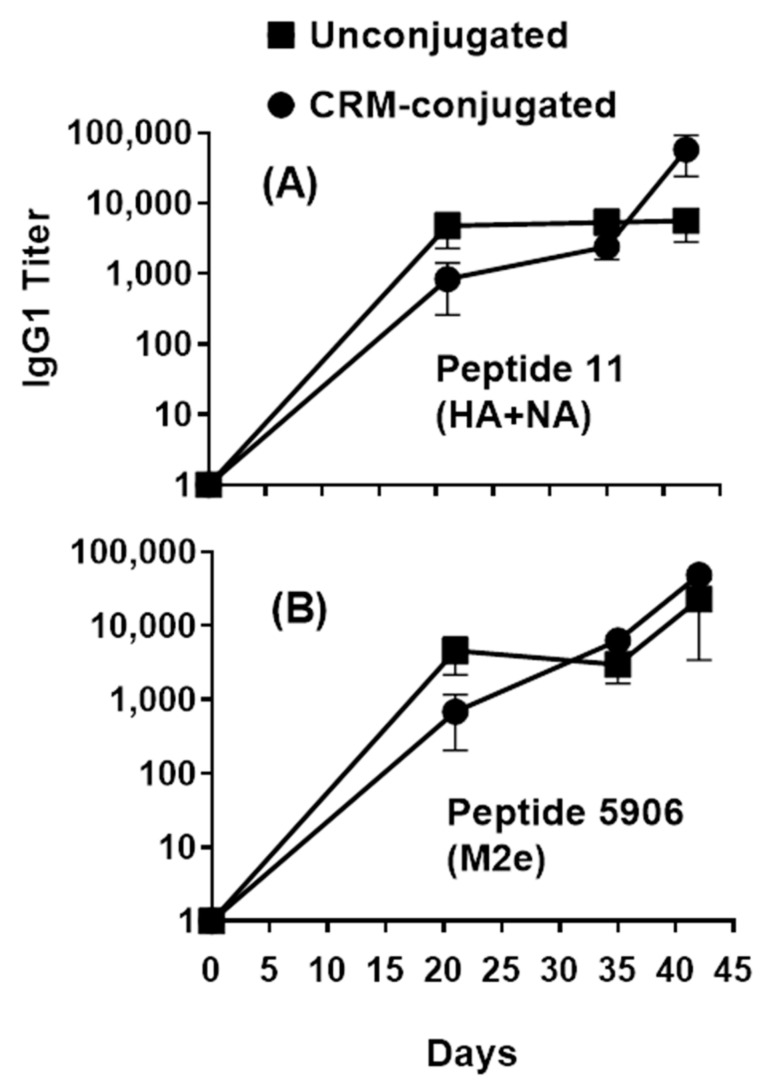
Profile of serum IgG1 responses across composite HA+NA and M2e peptides in mice immunized with 1 µg of composite influenza peptide (HA, NA and M2e) vaccine, unconjugated or CRM-conjugated, and formulated with ALFQ. Day 0, 21, 35, and 42 sera samples were analyzed by ELISA. Panel (**A**), IgG1 titers (1:100 dilution) on Flu Pep11 (HA+NA). Panel (**B**), on Flu Pep5906 (M2e). Data are expressed as mean ± SEM. Overall, the antibody responses on day 21 were significantly higher (ANOVA, *p* = 0.0421) for unconjugated peptides compared to CRM-conjugated peptides.

However, for CRM-conjugated mice, the IgG1 antibody responses were slightly higher later (Day 42) across composite HA+NA (Flu Pep11) and M2e (Flu Pep5906) conserved epitope (Figure 4). IgG2b antibody responses for unconjugated peptides were approximately 6-fold higher (on day 21) across conserved individual epitopes HA (Flu Pep3 and Pep6) and NA (Flu Pep10) with average titers of 217.0, 187.2, and 200.5, respectively compared to average titers of 33.6, 25.4, and 35.8, respectively, for the CRM-conjugated peptides. However, on day 42, the IgG2b antibody responses to HA (Flu Pep3 and Pep6) and NA (Flu Pep10) were approximately 1.5- to 2-fold higher for CRM-conjugated peptides compared to the unconjugated peptides (average titers of 1196.5, 406.5, and 2087.9 versus 642.1, 578.2, and 735.9, respectively). Across conserved composite epitopes HA+NA (Flu Pep11) and M2e (Flu Pep5906), the IgG2b antibody responses were approximately 2- to 5-fold higher (on day 21) for unconjugated peptides compared to CRM-conjugated peptides (average titers of 606.1 and 366.7 versus 115.4 and 133.5, respectively). The generation of IgG1 and IgG2b serum antibodies in both unconjugated and CRM-conjugated mice groups demonstrates the induction of both Th1 and Th2 immune responses.

**Figure 5:**
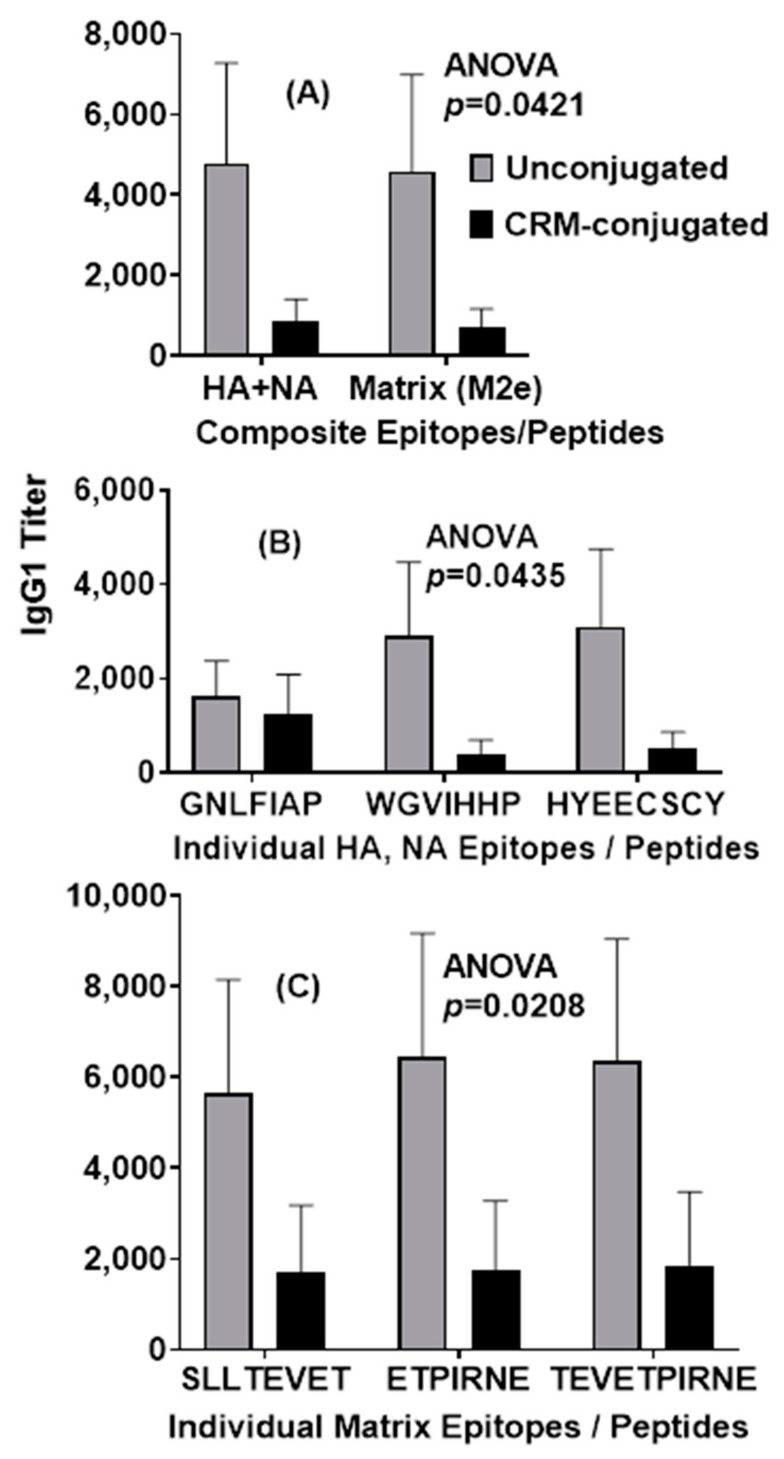
Mice immunized with 1 µg of unconjugated composite influenza peptide (HA, NA and M2e) vaccine formulated with ALFQ had significantly higher (ANOVA, *p* = 0.0421, *p* = 0.0435) IgG1 antibody responses 21 days after immunization when compared to CRM-conjugated composite peptide vaccines for both the composite influenza peptides and the individual influenza HA and NA peptide epitopes (Panels (**A**,**B**)). Serum IgG1 antibody responses to individual influenza M2e peptide epitopes were also significantly higher (ANOVA, *p* = 0.0208) in the unconjugated vaccine group on day 100 (Panel (**C**)). IgG1 titers (1:100 dilution) were analyzed by ELISA and expressed as mean ± SEM.

A statistical analysis of IgG1 serum antibody responses on day 21 revealed significant differences between the unconjugated and CRM-conjugated peptides across highly conserved composite HA+NA and Matrix (M2e) epitopes (*p* = 0.042) (Figure 5A) and across highly conserved individual HA and NA epitopes (*p* = 0.0435) (Figure 5B). Furthermore, across highly conserved individual matrix epitopes (M2e, M1 and M2), the IgG1 serum antibody responses also demonstrated significant differences (*p* = 0.0208) between the two groups on day 100 (Figure 5C).

### 3.2. Antisera Titers on Live Influenza A Viruses

**Figure 6:**
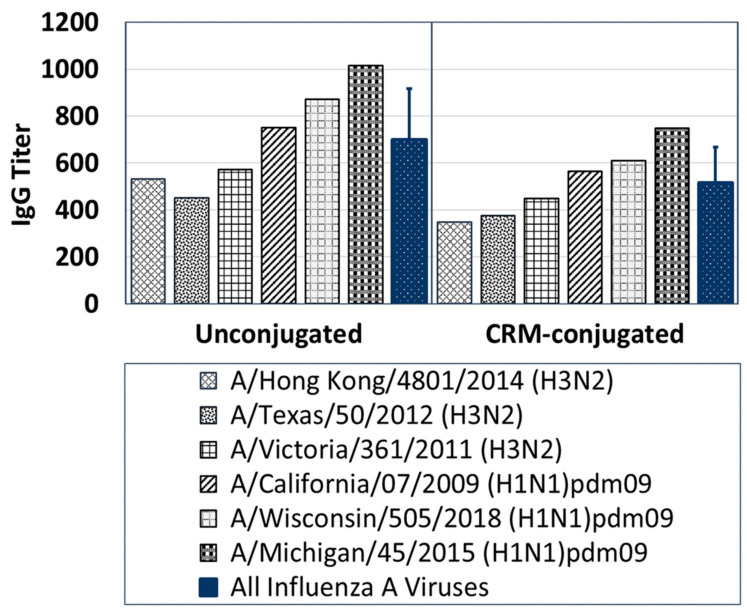
A comparison of IgG-specific antibody responses across several live contemporary influenza A H1N1 and H3N2 viruses for Day-56 serum samples (1:100 dilution) pooled from mice immunized with 1 µg of composite influenza peptide (HA, NA and M2e) vaccine, unconjugated or CRM-conjugated, and formulated with ALFQ. Average antisera titers (blue bars) for both unconjugated and CRM-conjugated mice groups were analyzed by ELISA and expressed as mean ± SD. Serum IgG antibody responses to Group 1 and 2 influenza viruses were higher (ns) in mice immunized with the unconjugated peptides compared to CRM-conjugated peptides.

Sera from mice immunized with composite peptide vaccine (NA, HA and M2e) at 1 µg/mouse demonstrated IgG antibody responses that bound broadly across several contemporary influenza A strains in Group 1 and Group 2 (Figure 6). Serum antibody titers to six live influenza A contemporary strains, although higher with the unconjugated peptide immunized mice group compared to the CRM-conjugated peptide mice group on day 56; the antibody responses showed no statistically significant differences between the two groups.

### 3.3. Serum Neutralizing Titers against Group 1 and 2 Influenza Viruses

Serum antibodies from both unconjugated and CRM-conjugated peptide vaccinated mice (1 µg/mouse) neutralized contemporary influenza A viruses in Group 1 and Group 2 to a similar extent (Table 2). For the unconjugated peptide-vaccinated mice groups, serum neutralizing titers ranged between 40–2560 (H1N1) and 1280–5120 (H3N2). In comparison, for the CRM-conjugated peptide-vaccinated mice groups, serum neutralizing titers ranged between 160–2560 (H1N1) and 1280–2560 (H3N2). No statistically significant differences were observed between the two groups (*p* > 0.5, Student’s *t*-test).

### 3.4. Cytokine Induction in Supernatant from Mouse Spleen Cells and Sera Samples

**Figure 7:**
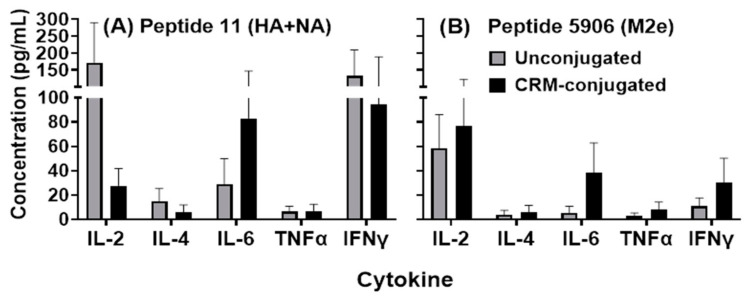
Analysis of cytokines produced by splenocytes harvested from mice injected with 1 µg of composite unconjugated or CRM-conjugated influenza peptide (HA, NA and M2e) vaccine formulated with ALFQ. Splenocytes were treated with 30 µg of each peptide, Flu Pep11 (HA+NA, Panel (**A**)) and Flu Pep5906 (M2e, Panel (**B**)), for 72 h and supernatant collected for analysis. Cytokines were analyzed using the Quansys Bioscience Q-Plex^TM^ Arrays. No significant differences were observed between the two groups (as determined by ANOVA). Data are represented as means ± SEM.

Cytokine induction was observed in supernatants from mice splenocytes incubated with HA, NA and M2e peptides in vitro (Figure 7A,B). Supernatants from mice immunized with 1 µg CRM-conjugated composite peptide had higher (not statistically significant) IL-6 levels in response to HA+NA (Flu Pep11) and M2e (Flu Pep5906) compared to mice immunized with the unconjugated peptides at a similar dose. Notably, in response to Pep11 (HA+NA) treatment, IL-2, IL-6, and IFN-gamma stimulation was evident in splenocytes from both unconjugated and CRM-conjugated peptide immunized mice; however, IL-2 stimulation was markedly higher (ns) in the unconjugated peptide immunized mice. Thus, both unconjugated and CRM-conjugated peptides induced Th1 and Th2 cytokines.

## 4. Discussion

One strategy for the development of peptide vaccines entails the incorporation of multiple copies of the same epitope to improve the humoral immune response to the target antigen. However, a more desirable approach that combines multiple epitopes from different key antigens could provide better coverage of pathogen antigen diversity and reduce the risk of pathogen escape due to immune pressure [24]. The HA, NA and matrix proteins provide a diverse set of highly conserved influenza virus epitopes that we incorporated into our composite influenza peptide immunogens. While HA has been a focus for many seasonal vaccines, other influenza proteins such as NA and M2e have been proposed as important vaccine components to induce broad neutralizing antibodies [5,6,7,8,9,10]. Monoclonal antibodies to each of the peptide epitopes in the composite antigens used in this study were shown to neutralize influenza viruses across Groups 1 and 2 contemporary strains (unpublished data). Additionally, the inclusion of a T-cell epitope may further promote broad immunity. Synthetic peptide vaccines eliminate the need for the cumbersome and time-consuming process of egg-based production, removing the possibility of antigenic changes during vaccine development [24,25].

Small peptide antigens are often poorly immunogenic unless they are conjugated to a carrier such as detoxified diphtheria or tetanus toxins. However, in this study we have shown that composite peptides comprising of small HA, NA and M2e highly conserved epitopes (conjugated and unconjugated) induced a robust antibody response when formulated with ALFQ adjuvant. As little as 1 µg of unconjugated composite peptide vaccine formulated with the ALFQ adjuvant induced broad influenza humoral immunity. Being able to induce high levels of peptide-specific antibodies without the need for conjugation simplifies the manufacturing process of peptide vaccines. Rapid induction of antibodies was observed after the initial immunization and both Th1 and Th2 immune responses were detected by antibody and cytokine analysis. These preliminary studies strongly suggest that both the targeted epitopes and the adjuvant formulation are important for induction of broadly neutralizing antibodies to influenza across Groups 1 and 2 influenza strains.

The ALF family of adjuvants has been used with a variety of antigens [22], and liposomes containing both MPLA and QS21 (AS01 adjuvant, GSK) are present in the first FDA-approved liposome-based vaccine, Shingrix, (GSK), a vaccine for the prevention of herpes zoster caused by a previous infection with varicella virus (chicken pox) [22]. ALFQ was shown to be safe with no adverse events in two different types of candidate malaria vaccines (unpublished data), and a phase 1 clinical trial with QS21 as the adjuvant component together with a spike ferritin nanoparticle construct for a COVID-19 vaccine is currently in progress. The unique formulation of influenza composite peptides and ALFQ provides stimulation of the immune system at many points. Previous studies have also shown that ALFQ provides a more balanced Th1 and Th2 response when compared to other liposomal adjuvants [22]. Moreover, the ability to promote broad immunologic stimulation with little or no apparent toxicity may allow enhanced immunity with very low levels of antigen.

Further immunogenicity studies are being conducted to analyze low-dose immunizations using various routes of administration. In addition, influenza challenge studies are being designed to evaluate protection against Group 1 and Group 2 influenza strains.

## 5. Conclusions

There is a need for improved influenza vaccines that include protection from both drifted and heterologous (pandemic) strains. A vaccine that incorporates a diverse set of conserved epitopes from HA, NA and M2e will likely bring us closer to that goal. While conjugation of small peptides to larger proteins improves peptide immunogenicity, adjuvants offer another key approach. ALFQ adjuvant played an important role in improving the immunogenicity of the composite influenza peptide vaccine and promoted the induction of a balanced Th1/Th2 response, while increasing its neutralization breadth against both Group 1 and Group 2 influenza viruses. In addition, formulation of composite peptides with ALFQ provided a dose-sparing effect that may decrease vaccine cost and improve production efficiency.

## Figures and Tables

**Table 1:** An overview of composite influenza peptides and their sequences comprising conserved HA, NA and M2e epitopes.

Composite Influenza Peptides	Unconjugated and Conjugated	
	Peptide Sequences	Conserved Epitopes
Flu Pep3	GNLFIAP	HA
Flu Pep6	WGVIHHP	HA
Flu Pep10	HYEECSCY	NA
Flu Pep11 ^1^	GNLFIAPWGVIHHPHYEECSCY	HA + NA
Flu Pep5906 ^2^	SLLTEVETPIRNEWGLLTEVETPIRQYIKANSKFIGITE	M2e + T-cell

^1,2^ These composite influenza peptides were used in both unconjugated and conjugated vaccine formulations with ALFQ.

**Table 2:** Influenza A neutralizing titers in Day-42 sera from ICR mice immunized with 1 µg of composite unconjugated or CRM-conjugated influenza peptide (HA, NA and M2e) vaccine formulated with ALFQ.

Influenza A Virus Strain		Serum Neutralizing Titers ^1^	
		Unconjugated	CRM-Conjugated
	A/Hong Kong/4801/2014 (H3N2)	1280	1280
**H3N2**	A/Texas/50/2012 (H3N2)	2560	2560
	A/Victoria/361/2011 (H3N2)	5120	1280
	A/Michigan/45/2015 (H1N1)pdm09	2560	2560
**H1N1**	A/California/07/2009 (H1N1)pdm09	40	320
	A/Wisconsin/505/2018 (H1N1)pdm09	320	160

^1^ Serum neutralizing titers across Group 1 and 2 viruses (H1N1 and H3N2) were calculated using the microneutralization assay. No statistically significant differences were observed (as determined by Student’s t-test) between the unconjugated and CRM-conjugated peptide immunized mice groups.

## Data Availability

The data are available from the corresponding author upon request.
